# Supplementary material for: FKBP5 rs4713916: A Potential Genetic Predictor of Interindividual Different Response to Inhaled Corticosteroids in Patients with Chronic Obstructive Pulmonary Disease in a Real-Life Setting
Source: Int J Mol Sci. 2019 Apr 24;20(8):2024. doi: 10.3390/ijms20082024 (PMC6514776; doi:10.3390/ijms20082024)
Supplement: Supplementary file 1 [file ijms-20-02024-s001.pdf]

# Supplementary Figures

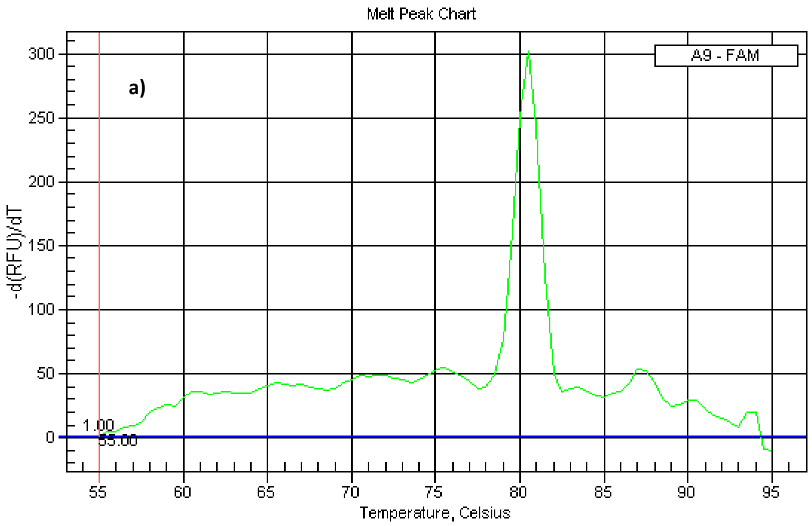


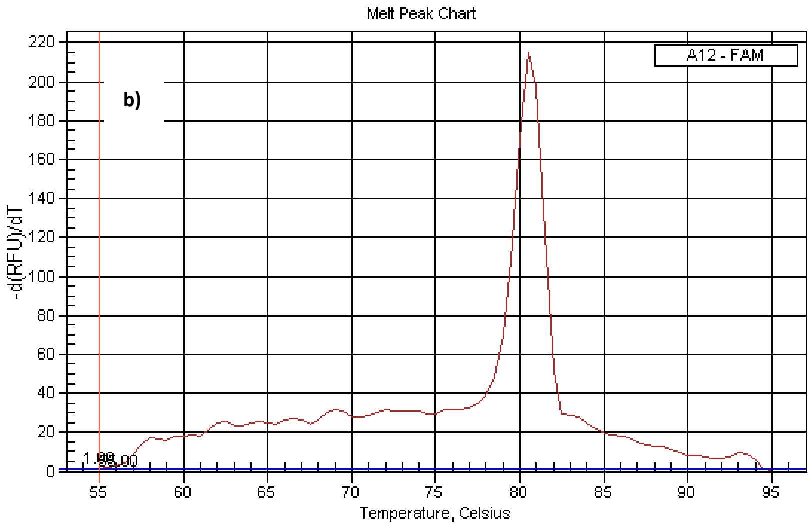


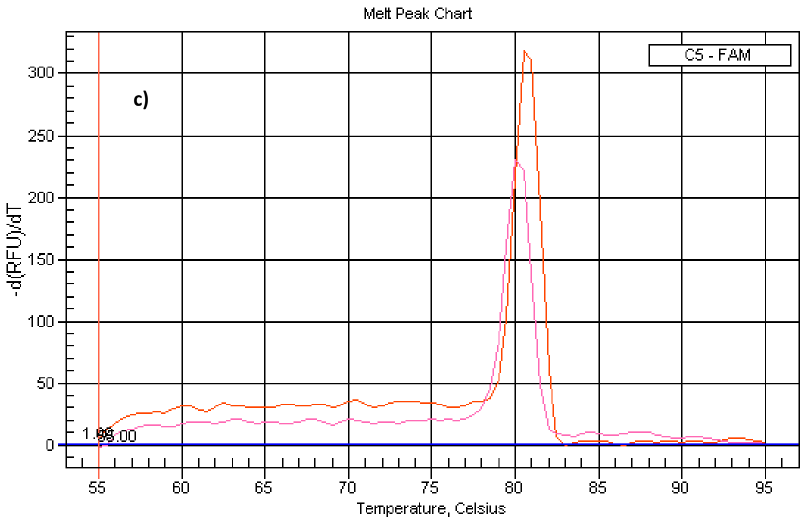

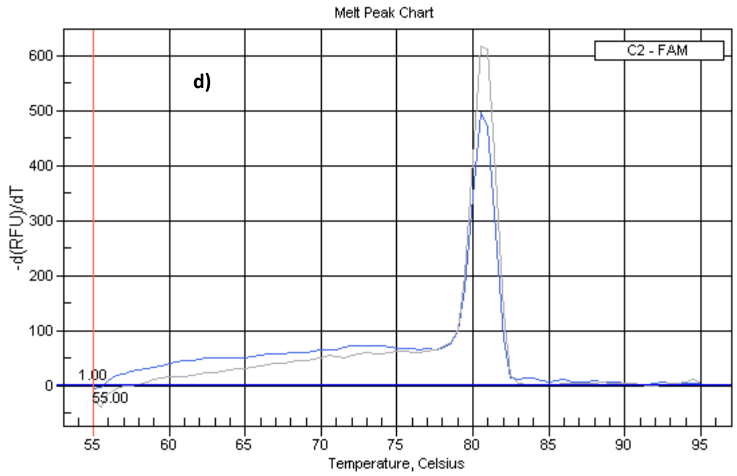


**Figure S1.** Melting profile for the allele variants of MDR1 polymorphism (rs2032582) using allele-specific PCR method. (a): genotype GG; (b): genotype TT; (c): genotype GT; (d): genotype GA.

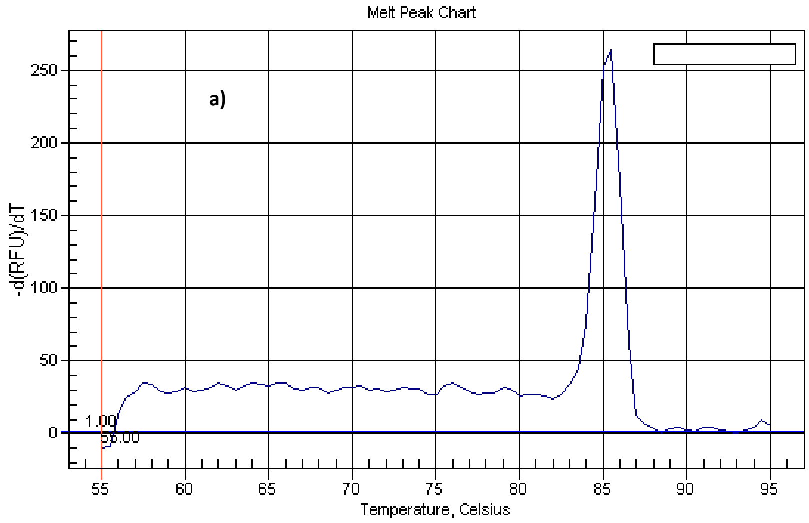

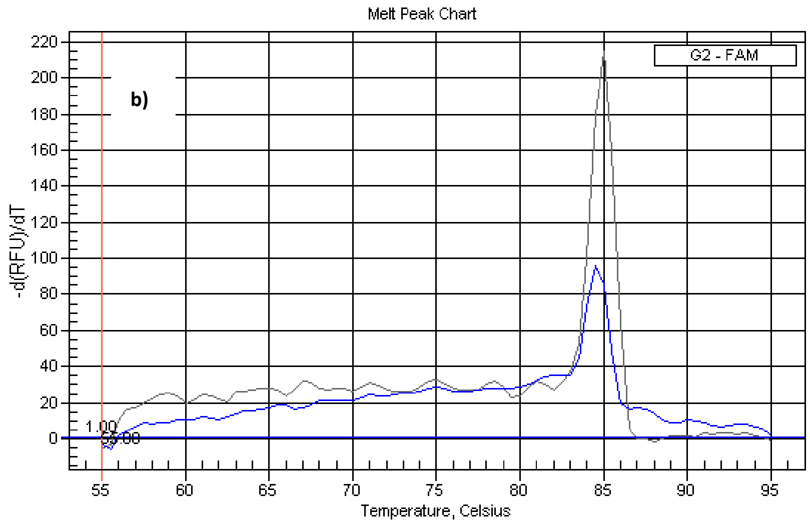


**Figure S2.** Melting profile for the allele variants of NR3C1 polymorphism (rs6189) using allele-specific PCR method. (a): genotype GG; (b): genotype GA.

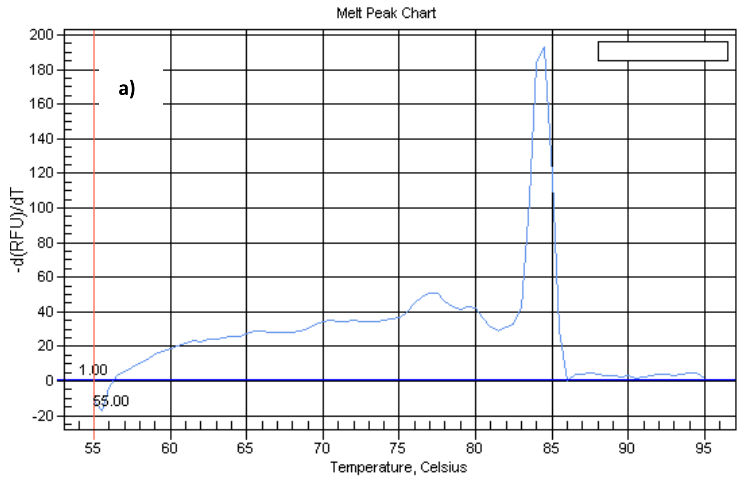

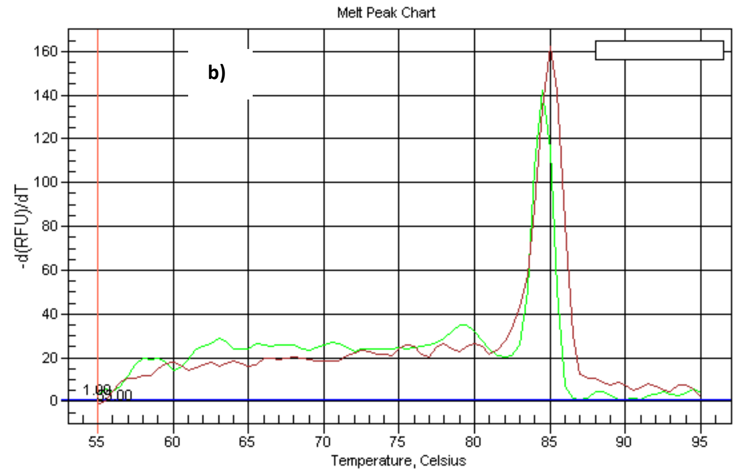


**Figure S3.** Melting profile for the allele variants of NR3C1 polymorphism (rs6190) using allele-specific PCR method. (a): genotype GG; (b): genotype GA.

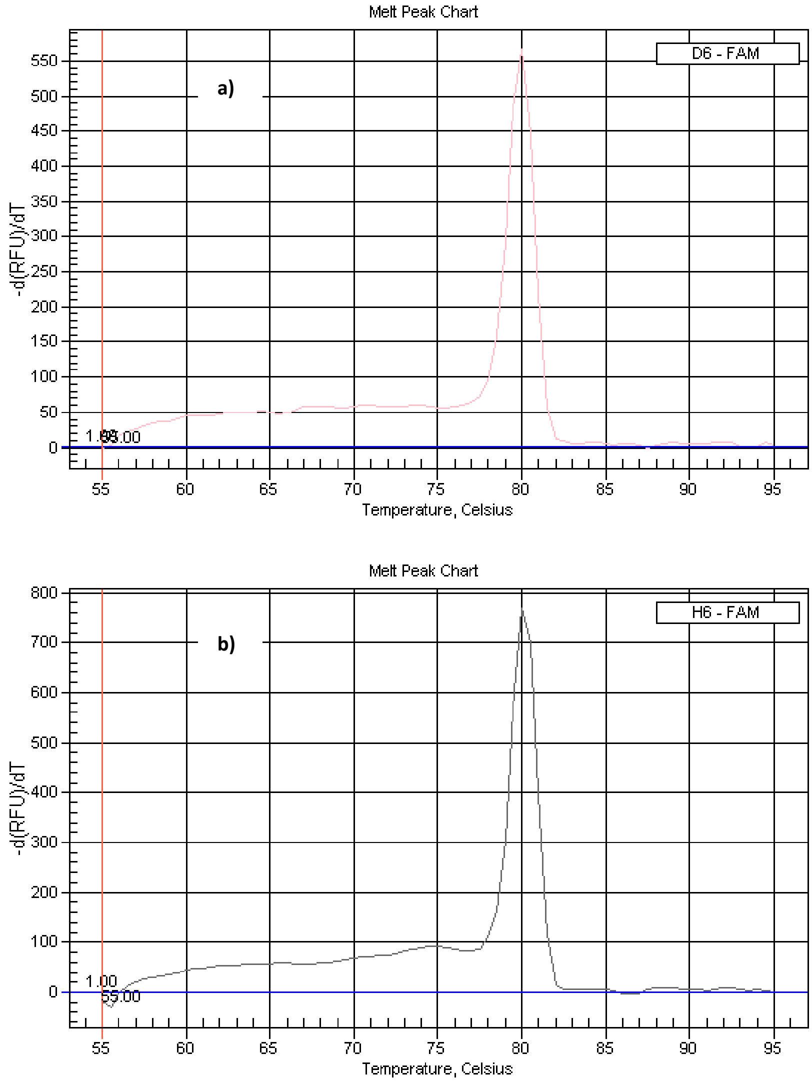

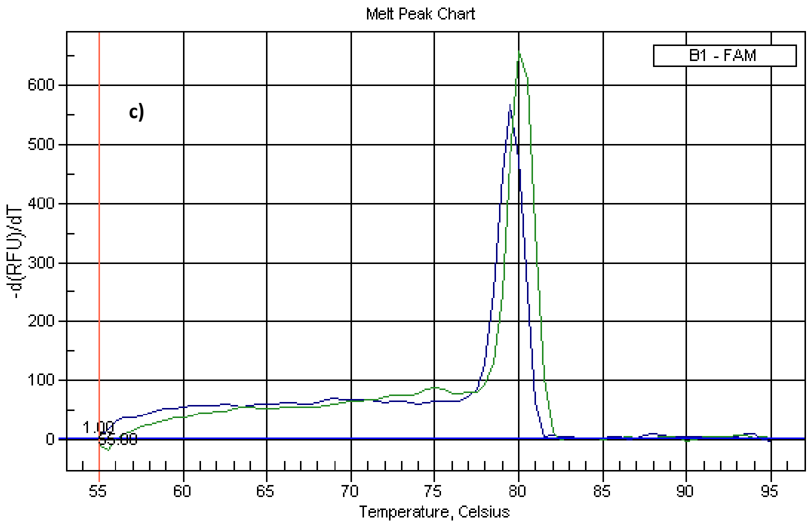


**Figure S4.** Melting profile for the allele variants of NR3C1 polymorphism (rs41423247) using allele-specific PCR method. (a): genotype GG; (b): genotype CC; (c): genotype CG.

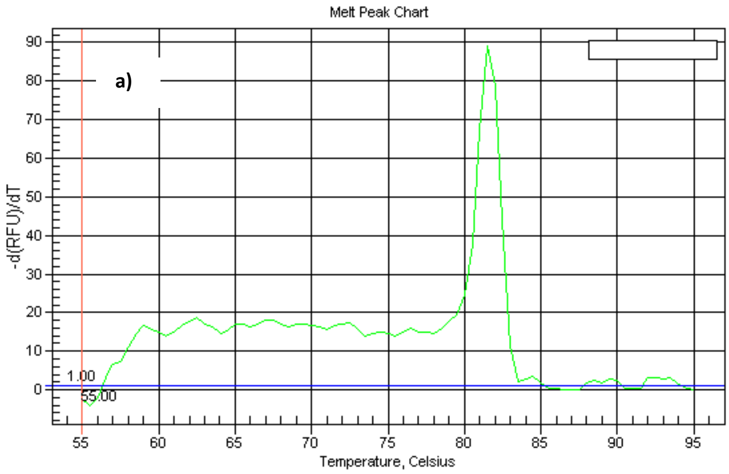


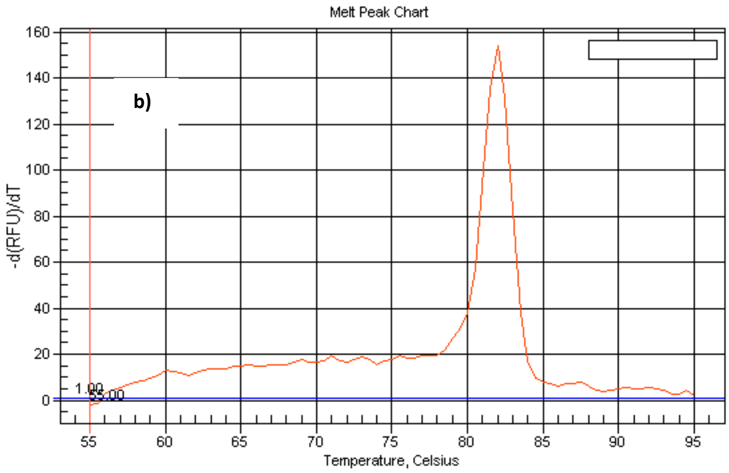

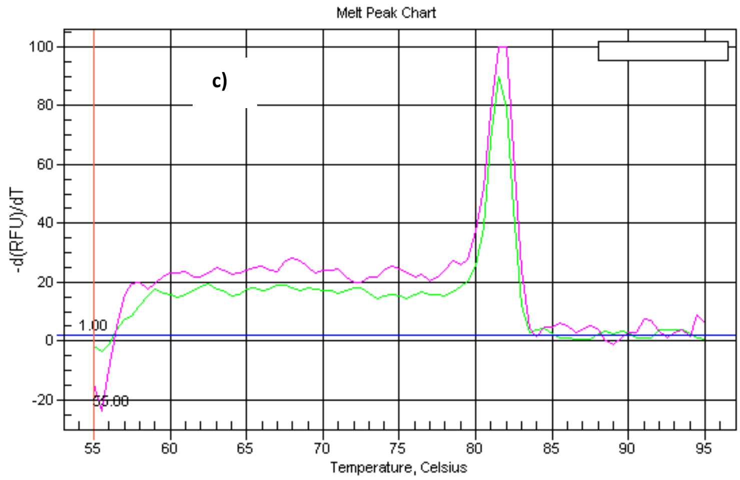


**Figure S5.** Melting profile for the allele variants of GLCCI1 polymorphism (rs37972) using allele-specific PCR method. (a): genotype CC; (b): genotype TT; (c): genotype CT.

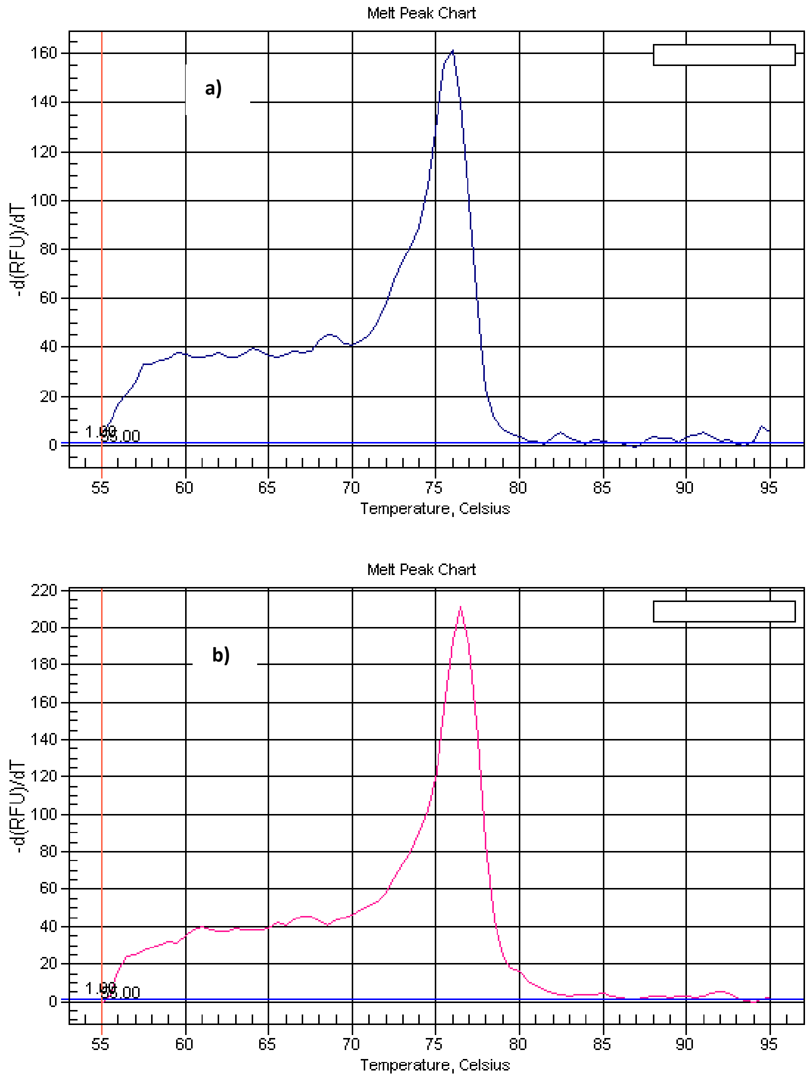

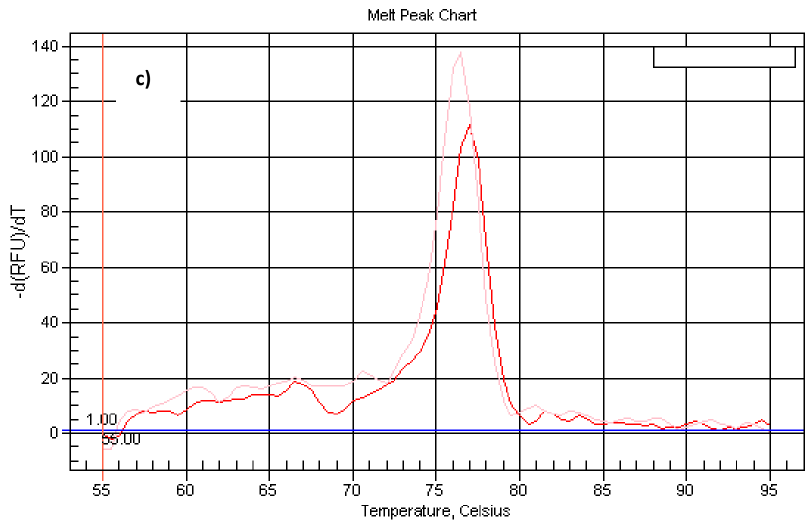


**Figure 6.** Melting profile for the allele variants of FKBP5 polymorphism (rs41423247) using allele-specific PCR method. (a): genotype AA; (b): genotype GG; (c): genotype GA.
